# Supplementary material for: Emerging Perspectives on the Rare Tubulopathy Dent Disease: Is Glomerular Damage a Direct Consequence of ClC-5 Dysfunction?
Source: Int J Mol Sci. 2023 Jan 9;24(2):1313. doi: 10.3390/ijms24021313 (PMC9864126; doi:10.3390/ijms24021313)
Supplement: Supplementary file 1 [file ijms-24-01313-s001.zip › ijms-2084839-supplementary.pdf]

Supplementary Figure

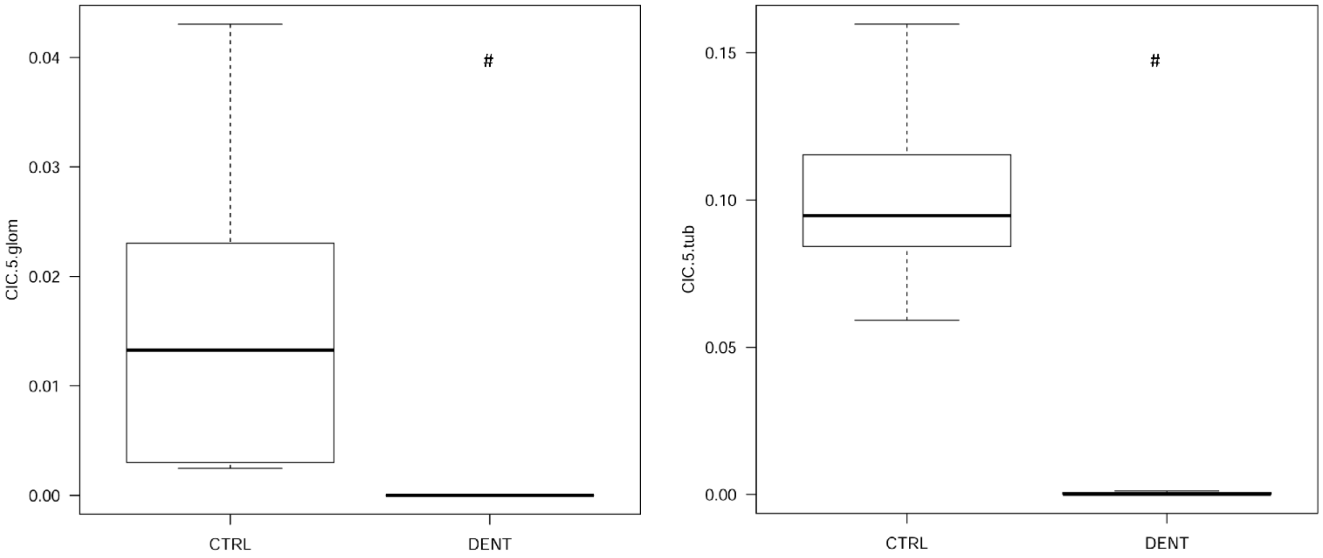

Figure S1. **Morphometric evaluation of ClC-5 in CTRL and DD1 biopsies in both tubular and glomerular compartments**. ClC-5 resulted down-regulated in DD1 vs CTRL (#p<0.01) in both glomerular and tubular compartments. Boxplots show medians and interquartile ranges of IHC staining scores (% positive area).

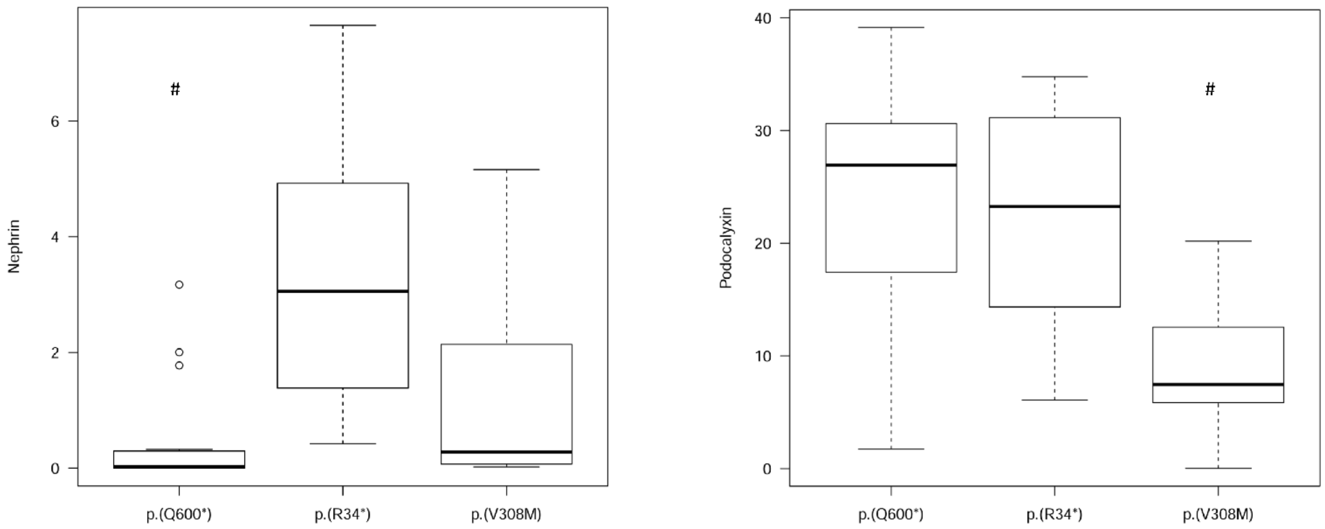

Figure S2. **Morphometric evaluation of nephrin and podocalyxin in DD1 biopsies from patients carrying different *CLCN5* mutations**. The patient carrying the p.(Q600*) mutation shows the highest down-regulation of Nephrin (#p<0.01). The patient carrying the p.(V308M) mutation shows the highest down-regulation of Podocalyxin (#p<0.01). The lowest change in both markers was seen in the patient carrying the p.(R34*) mutation. Boxplots show medians and interquartile ranges of IF staining scores (% positive area).

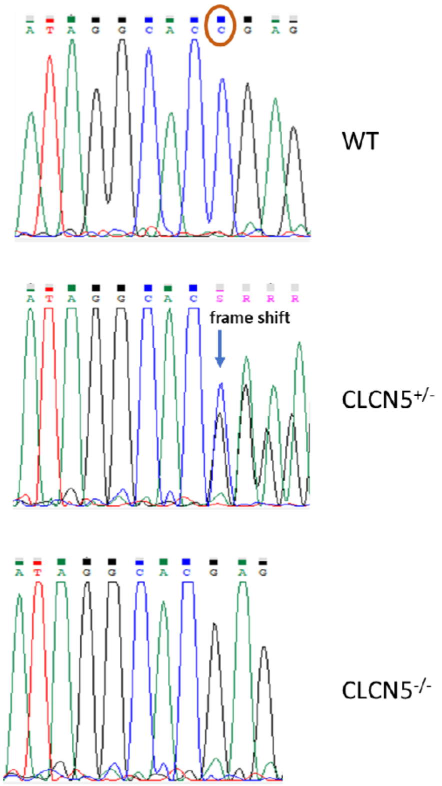

Figure S3. ***CLCN5* gene editing in podocyte cells by CRISPR-Cas9 technology**. Representative image of Sanger sequencing of PCR products of exon 2 from WT, CLCN5$^{+/-}$ and CLCN5$^{-/-}$ clones. The c.100delC mutation is shown.

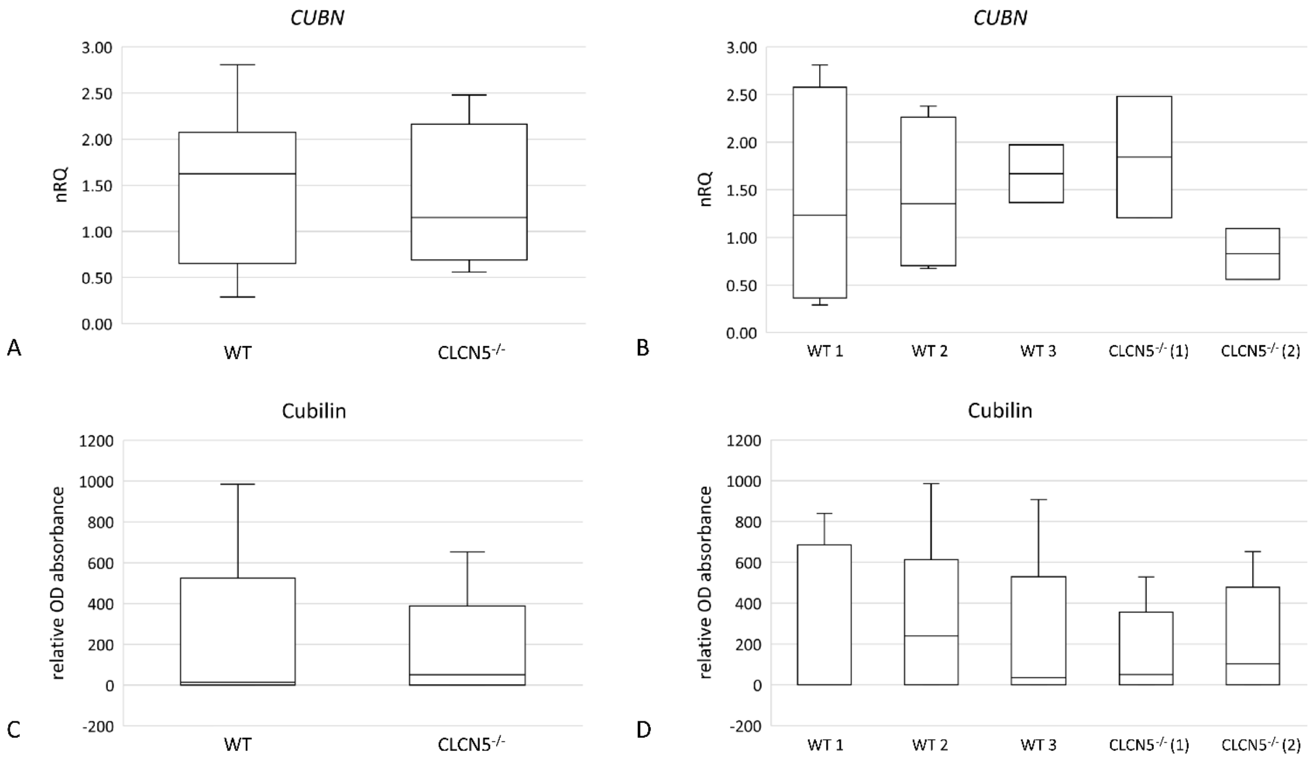

Figure S4. Cubilin gene (*CUBN*) and protein expression in WT and *CLCN5* $^{-/-}$ clones. Boxplots show medians and interquartile ranges of (**A-B**) relative mRNA, as determined by qRT-PCR, and (**C-D**) protein expression, as determined by ICW. p-values were obtained with the Mann-Whitney U test. Results are from two independent experiments performed in triplicate. Abbreviations: nRQ: normalized relative quantity; OD, optical density.
